# Pseudoaneurysm of mitral-aortic intervalvular fibrosa with rupture: a case report

**DOI:** 10.1186/s40792-023-01789-3

**Published:** 2023-12-04

**Authors:** Kosuke Nakata, Shuji Moriyama, Jun Takaki, Masahiro Takeo, Hideki Doi, Toshiyuki Matsumura, Toshihiro Fukui

**Affiliations:** 1 Department of Cardiovascular Surgery, Kumamoto Rosai Hospital, 1670 Takehara-Machi, Yatsushiro, Kumamoto 866-8533 Japan; 2 Department of Cardiology, Kumamoto Rosai Hospital, Yatsushiro, Kumamoto Japan; 3 Department of Cardiovascular Surgery, Kumamoto University Hospital, Kumamoto, Japan

**Keywords:** Mitral-aortic intervalvular fibrosa, Pseudoaneurysm, Rupture, Infective endocarditis, Intracranial hemorrhage

## Abstract

**Background:**

Mitral-aortic intervalvular fibrosa (MAIVF) is a fibrous region connecting the anterior mitral leaflet (AML) and aortic valve. Pseudoaneurysm of the MAIVF is a rare condition that has been reported as a sequela of infective endocarditis (IE) and surgical trauma. Here, we report a case of a ruptured pseudoaneurysm of the MAIVF, along with some literature reviews.

**Case presentation:**

A 65-year-old man diagnosed with moderate aortic regurgitation five years previously had a fever of unknown origin. He suddenly developed headache and apraxia and was transported to our hospital. He was diagnosed with intracranial hemorrhage and admitted. One week after admission, echocardiography revealed aorto-mitral discontinuity and protrusion with severe regurgitant flow from left ventricular outflow tract to the left atrium. The AML was suspected to have ruptured. However, intraoperatively, the AML structure was preserved. A ruptured pseudoaneurysm of the MAIVF was also observed. Therefore, we successfully performed pseudoaneurysm repair using a bovine pericardial patch, aortic valve replacement, and mitral annuloplasty.

**Conclusions:**

P-MAIVF is a rare but potentially life-threatening complication of IE, for which timely diagnosis and prompt appropriate therapeutic intervention are required. In the present case, although neither obvious active IE nor history of previous IE could be identified, healed IE was considered based on the clinical course. The patient had intracranial hemorrhage (ICH) with well-controlled heart failure and underwent elective surgical repair more than one month after the onset of ICH, while the clinical course after the surgical procedure was uneventful.

## Background

Mitral-aortic intervalvular fibrosa (MAIVF) is a fibrous region connecting the anterior mitral leaflet (AML) and the aortic valve [1]. Pseudoaneurysm of the MAIVF (P-MAIVF) is a rare condition that has been reported as a sequela of infective endocarditis (IE) and surgical trauma [2]. Additionally, neurologic complications, such as strokes, intracranial hemorrhage (ICH), mycotic aneurysms, and cerebral abscess, are common in patients with IE [3]. We report a case of ruptured P-MAIVF with ICH in which the involvement of aortic valve regurgitation (AR) and IE was suspected, with some literature reviews.

## Case presentation

**Fig. 1:**
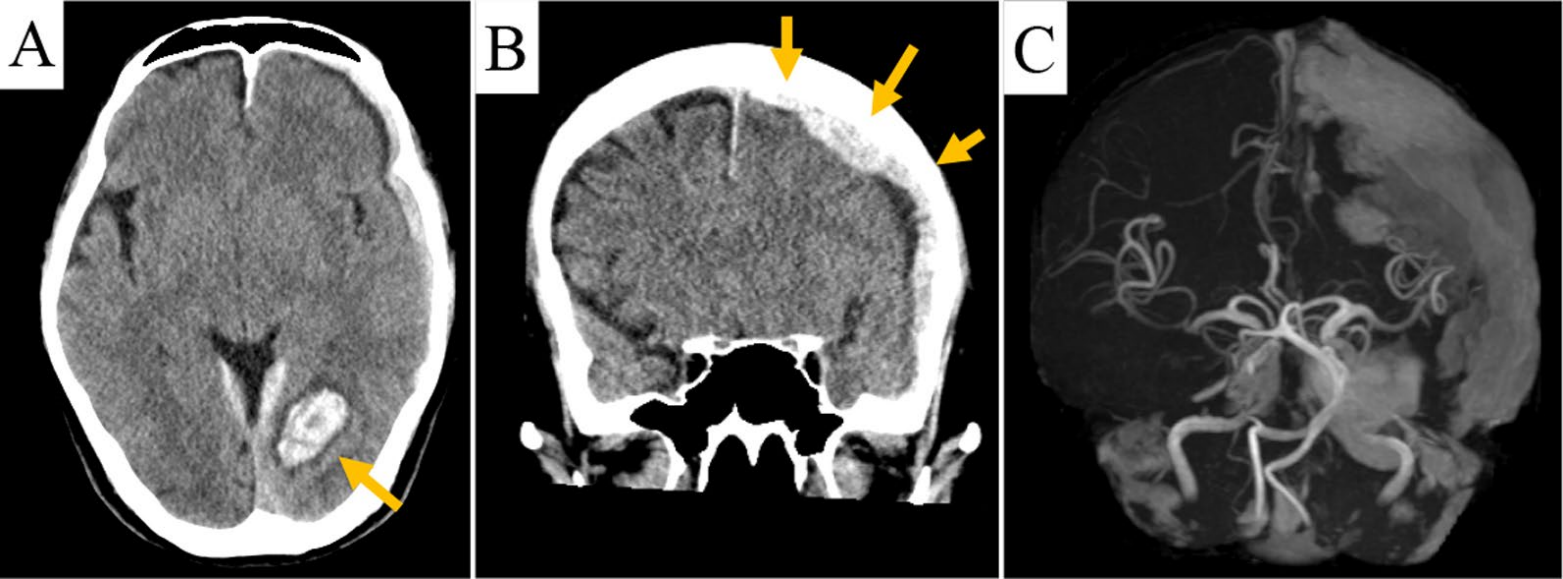
**A**, **B** Computed tomography showed a left acute subdural hematoma and hemorrhage, and a left subcortical occipital lobe hemorrhage. **C** Magnetic resonance angiography did not reveal any blockage of the major vessels, and no mycotic aneurysms were observed

**Fig. 2:**
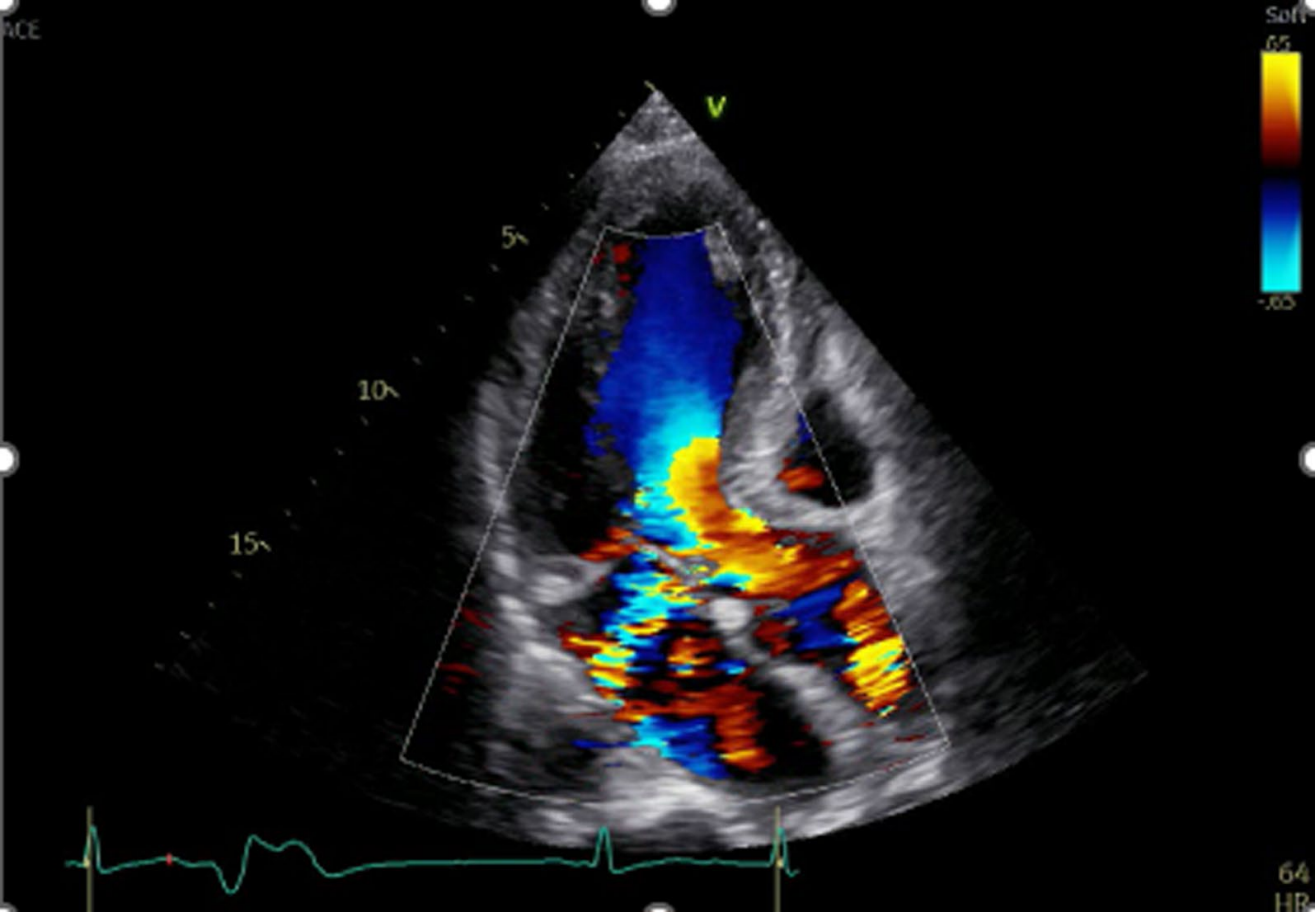
Transthoracic echocardiography revealed the pseudoaneurysm and a backflow into the left atrium through the midline of the anterior mitral leaf (AML). There was moderate aortic regurgitation (AR) and the direction of the AR jet faced to the pseudoaneurysm

**Fig. 3:**
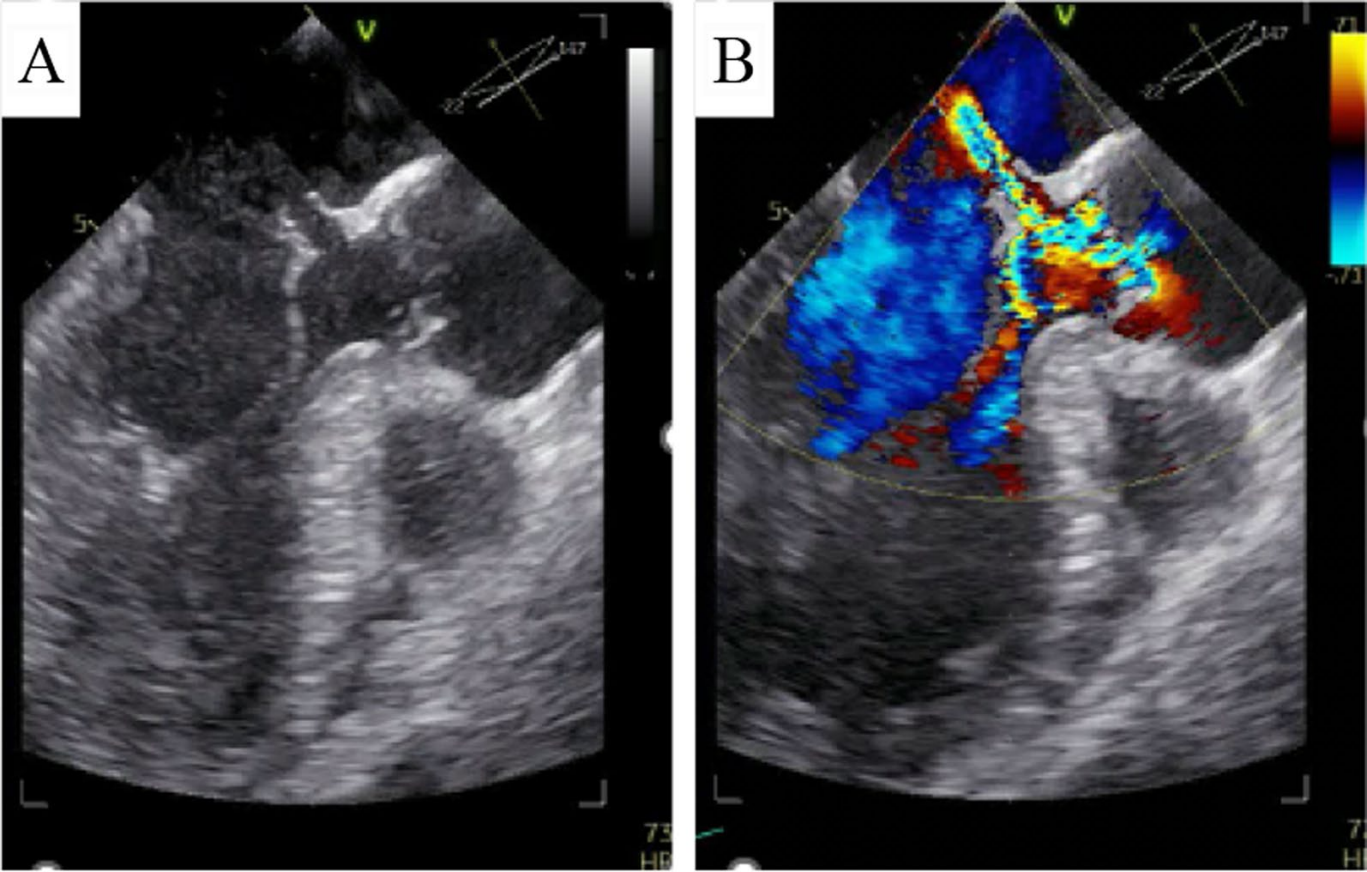
**A** Transesophageal echocardiography (TEE) showed the pseudoaneurysm with a perforation at the AML. **B** TEE revealed moderate AR due to right coronary cusp prolapse and severe regurgitant flow from left ventricular outflow tract to the left atrium in systole

A 65-year-old man with headache and apraxia was referred to the emergency department of our hospital. The patient had been followed up for asymptomatic moderate AR for more than five years. He presented with fever (> 38 °C) and fatigue lasting for two weeks. He received antibiotic treatment for two weeks at another hospital. On physical examination, his arterial pressure was 150/70 mmHg. Although he had apraxia, he was conscious and alert, without motor aphasia on neurological examination. He had a diastolic cardiac murmur at the left parasternal border. There were no specific risk factors for IE, including drug abuse or history of dental procedures. Laboratory data showed an elevated C-reactive protein level (1.56 mg/L; reference value: 0.0–0.14 mg/L) and neutrophilic (92.6%) leukocytosis (20,800/µL; reference value: 3300–8600). Brain computed tomography and magnetic resonance imaging revealed subcortical hemorrhage in the left occipital lobe with subdural hemorrhage (Fig. 1A, B). Magnetic resonance angiography did not reveal any blockage of the major vessels, and no mycotic aneurysms were observed (Fig. 1C). Chest radiography revealed cardiomegaly, without pulmonary congestion. Transthoracic echocardiography (TTE) in the emergency room showed a mildly dilated and well-contracting left ventricle (left ventricular ejection fraction: 60%). Moderate perivalvular AR with eccentric jet to the MAIVF caused by prolapse of the right coronary cusp was observed, although mitral valve regurgitation was trivial. In addition, the TTE showed no evidence of vegetation. He was admitted to the neurosurgical department with an intracranial hemorrhage and subdural hematoma. The patient’s neurological symptoms gradually improved. However, one week after admission, TTE and transesophageal echocardiography (TEE) showed aorto-mitral discontinuity and protrusion with severe regurgitant flow from left ventricular outflow tract (LVOT) to the left atrium (Figs. 2, 3A and B). Absence of spike fever and negative blood culture results were observed after admission. There was not any sign of IE. Based on these findings, antibiotic treatment for neutrophilic leukocytosis was not administered. Thus, the patient was referred to us for surgical treatment. However, since the patient was not only accompanied with ICH but also stable hemodynamics, we decided to delay surgery.

**Fig. 4:**
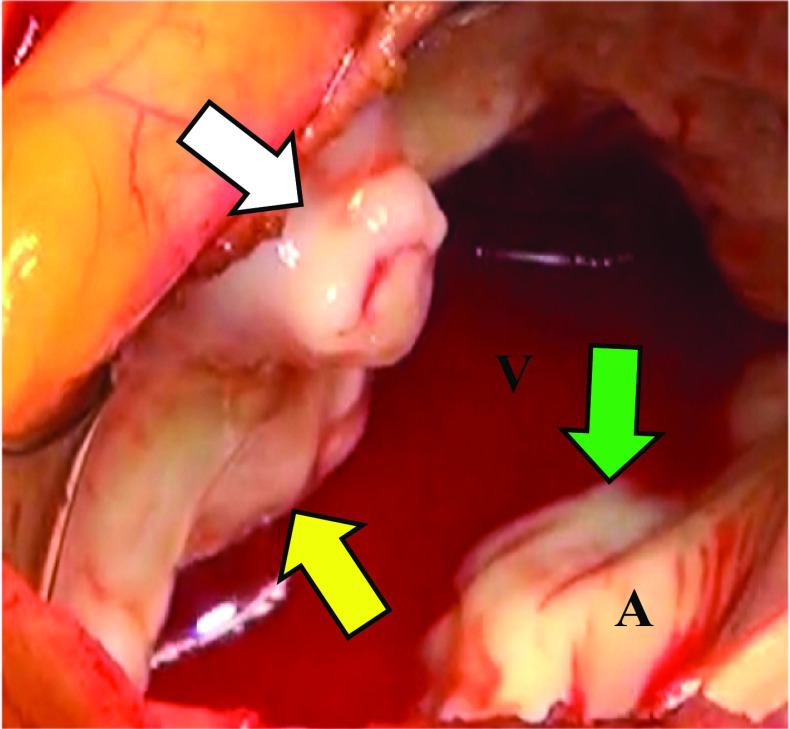
View from left atrial side. There was approximately 10 mm pseudoaneurysm of mitral-aortic intervalvular fibrosa with rupture (white arrow) but no obvious vegetation around the aortic valve and mitral valve (yellow arrow: AML, green arrow: posterior mitral leaf). A = left atrial side. V = left ventricular side

**Fig. 5:**
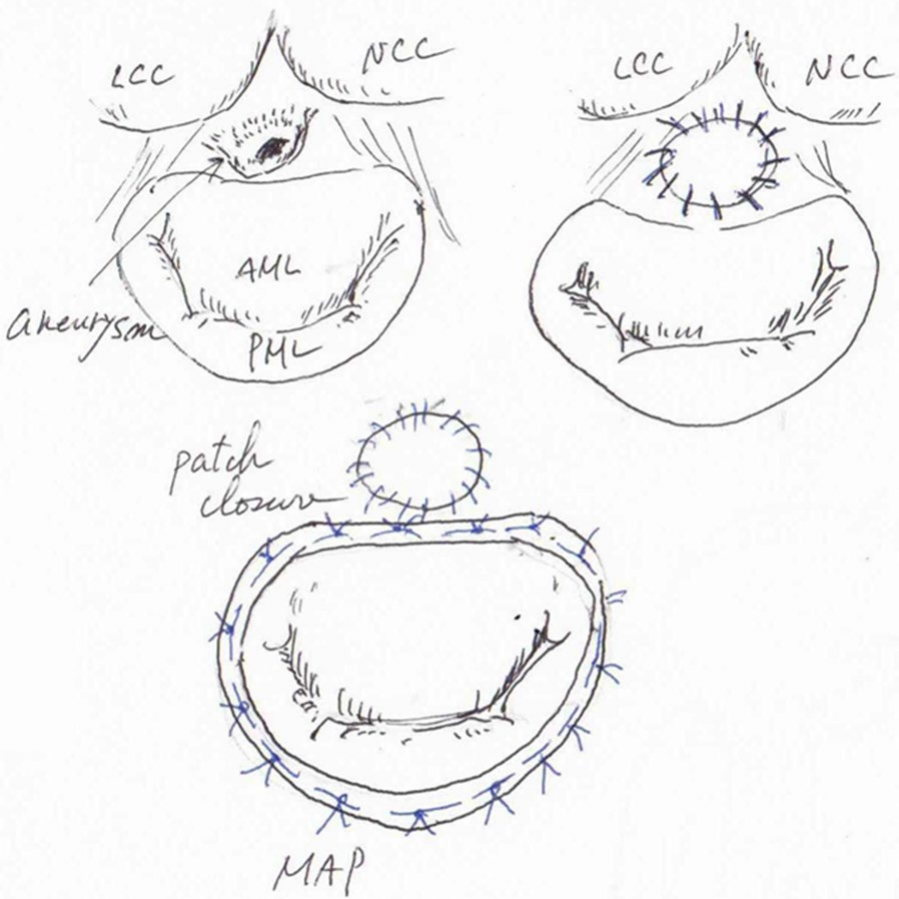
Surgical schema

The patient successfully underwent aortic valve replacement (AVR) with a 23-mm INSPIRIS RESILIA aortic heart valve (Edwards Lifescience, CA., USA) and mitral valve annuloplasty with a 26-mm MEMO 4D (LivaNova, London, UK) with P-MAIVF repair more than four weeks after the onset of ICH. Intraoperatively, there was no defect of AML but we found a rupture of P-MAIVF near the middle of the MAIVF. P-MAIVF was formed from LVOT toward left atrial side. There was no obvious vegetation in the cardiac chamber (Fig. 4). The procedure consisted of ruptured P-MAIVF removal and closure by a bovine pericardial patch (Edwards Lifescience, CA., USA) (Fig. 5). The histological findings of both removed aortic valve cusps and aneurysmal change with a perforation of MAIVF indicated myxoid degeneration without infiltrations of inflammatory cells and bacterial bodies. In addition, the bacteriological examination revealed no evidence of bacterial infection. The post operative TTE showed no evidence of vegetations, mitral valve regurgitation, and regurgitant flow from left ventricular to the left atrium.

The clinical course following the surgical procedure was uneventful, while the patient was discharged 15 days after surgery. The patient was followed without complications.

## Discussion

The MAIVF is a thin membranous structure between the half of the noncoronary cusp, a third of the left coronary cusp of the aortic valve, and the AML [2]. The MAIVF is composed of the pericardium, while its ventricular side forms by the LVOT [2]. Since the MAIVF is relatively avascular with offering little resistance to infection, infection of this area may result in the formation of abscesses or pseudoaneurysms, or a direct communication from the LVOT to the left atrium [1]. These damages can contribute to the P-MAIVF associated with fatal complications [2]. Furthermore, the P-MAIVF is a rare condition that has been reported as a sequela of infective endocarditis (IE) and surgical trauma [4]. Şahan et al. reported that 40% of patients with the P-MAIVF had active endocarditis and that 18% had a history of previous endocarditis in 166 cases of their literature review [2]. Sudhakar et al. reported that 72% of patients with the P-MAIVF who had histories of active or prior endocarditis in 89 cases of their literature review [4]. In addition, they reported that prosthetic aortic valves and histories of endocarditis were at high risk for developing the P-MAIVF [4].

Although the P-MAIVF often remains asymptomatic in absence of complications, the most frequent presentation was symptoms and/or signs of infection from active endocarditis, followed by dyspnea and heart failure [2, 4]. If the P-MAIVF progressively enlarges and compresses around structures, the patient presents with a variety of symptoms. Compression of the coronary arteries leads to angina or myocardial infarction. The left circumflex coronary artery is often compressed because of its anatomical relationship with its position [4]. Compression of the mitral valve causes mitral valve deformity and mitral regurgitation [5]. Rupture into the pericardial may cause pericardial tamponade [6]. As in our case, perforation or rupture of the P-MAIVF can lead to regurgitation from the LVOT to the left atrium. Echocardiographics revealed an eccentric jet mimicking mitral regurgitation (Figs. 2, 4).

Differential diagnoses include aortic valve annulus abscess, mitral regurgitation, anterior mitral valve aneurysm, and ruptured aneurysm of Valsalva [2, 7]. Initially, our patient was suspected to have an anterior mitral valve aneurysm. However, intraoperative findings showed P-MAIVF.

Echocardiography is useful for the diagnosis of P-MAIVF. TEE is superior to TTE in detecting cavity lesions in MAIVF. Afridi et al. reported that the diagnostic sensitivity of TEE was higher than that of TTE (43% vs. 90%, respectively; *P* < 0.01) [8]. An important echocardiographic finding for diagnosis of P-MAIVF is a pulsatile echo-free sac that expands in systole and collapses in diastole [2]. This finding provides clues about presence of P-MAIVF [6].

The patient had asymptomatic moderate AR with eccentric regurgitant jet for approximately five years previously. He had been evaluated by TTE for AR without significant changes. He had no previous history of cardiovascular intervention. His clinical records suggested episodes of IE, such as a recent history of fever of unknown origin and current ICH of unknown etiology. Therefore, we suspected that the patient may have presented with IE. However, bacterial and laboratory examinations, intraoperative findings, and histological findings didn’t indicate any current infection.

We considered that AR and endocarditis contributed to ruptured P-MAIVF. We hypothesized that a mechanism of ruptured P-MAIVF was as follows. We suspected that he was the IE by clinical course. Because the infection was spread to the MAIVF, these tissues was destroyed and weakened. The continuous eccentric AR jet hit these weaken area and added a physical load. As a result, the pseudoaneurysm and perforation was formed in short-term. Aortic regurgitation is a contributing factor by adding further insult to the already compromised MAIVF region by the regurgitant jet blood flow as well as by helping establish a secondary infected site in this region [9]. Transthoracic echocardiography and TEE showed the blood flow of the AR jet directly crashing into the P-MAIVF in Figs. 2 and 3, respectively. Because of the rapid formation of P-MAIVF after admission, it is plausible that the fragile P-MAIVF by the continuous blood flow of the AR jet may have become infected and brittle, and then ruptured owing to the additional mechanical stress caused by the AR jet. Postoperative pathological findings showed no obvious active infectious findings such as infiltration of inflammatory cells. Microbiological findings of the resected tissues and blood culture results were negative. Therefore, the patient was considered to have recovered from IE.

The natural course of uncomplicated P-MAIVF remains unclear. In addition, it is well known that left ventricular pseudoaneurysms are prone to rupture. [10] Therefore, surgical repair is currently the recommended treatment to prevent further enlargement and complications [4]. Aortic valve replacement was performed in most patients in conjunction with some type of pseudoaneurysm repair, simple closure of the aneurysmal mouth, patch closure using a pericardial or synthetic graft, and aortic root replacement [2, 4, 11]. The patient was underwent AVR and mitral valve repair with P-MAIVF repair. We approached via transseptal which provide a good visual field. We resected P-MAIVF and patch reconstruction with bovine pericardial patch (Edwards Lifescience, CA., USA) through transseptal approach to prevent re-rupture because tissues of P-MAIVF was weakened. The bovine pericardial patch was made to fit the resection area with 3 mm extra margin for suture. Carpentier et al. said that associated aortic valve involvement requires a combined technique of valve replacement and annular reconstruction [12]. Therefore, we performed MAP for reinforcement of mitral valve annulus. The patch did not affect the procedure of MAP (Fig. 5). We selected a bioprosthetic valve for AVR because of a history of ICH.

Although the patient had volume overload from left ventricular to left atrial, similar to MR, there was no sign of heart failure. Therefore, according to the guidelines [3, 13, 14], the patient underwent surgical repair more than four weeks after the onset of ICH, whereas the clinical course following the surgical procedure was uneventful.

## Conclusion

P-MAIVF is a rare but potentially life-threatening complication of IE. The management of P-MAIVF requires detailed analysis of complications and imaging findings. In addition, timely diagnosis and prompt appropriate therapeutic intervention are required. In the present case, although neither obvious active IE nor history of previous IE could be identified, healed IE was considered based on the clinical course. The patient had ICH with well-controlled heart failure and underwent elective surgical repair more than one month after the onset of ICH, while the clinical course after the surgical procedure was uneventful.

## Data Availability

None.

## Abbreviations

AML: Anterior mitral leaflet
AR: Aortic valve regurgitation
AVR: Aortic valve replacement
ICH: Intracranial hemorrhage
IE: Infective endocarditis
LVOT: Left ventricular out flow tract
MAIVF: Mitral-aortic intervalvular fibrosa
p-MAIVF: Pseudoaneurysm of the mitral-aortic intervalvular fibrosa
TEE: Transesophageal echocardiography
TTE: Transthoracic echocardiography
